# Heat stress suppresses DNA replication and mitosis in barley root apical meristems

**DOI:** 10.3389/fpls.2025.1679234

**Published:** 2025-11-27

**Authors:** Kateřina Kaduchová, Peter Šály, Ivan Kashkan, Ales Pecinka

**Affiliations:** 1 Centre of Plant Structural and Functional Genomics, Institute of Experimental Botany (IEB), Czech Acad Sci, Olomouc, Czechia; 2 Department of Cell Biology and Genetics, Faculty of Science, Palacký University, Olomouc, Czechia; 3 Imaging Facility, Institute of Experimental Botany (IEB), Czech Acad Sci, Praha, Czechia

**Keywords:** *Hordeum vulgare*, heat stress, mitosis, cell cycle, root growth, chromatin, live-cell imaging, crops

## Abstract

Increasing temperature affects plant development, with the assumption that roots are among the tissues particularly sensitive to heat stress (HS). However, a comprehensive analysis of the impact of high temperature on the dynamics of cell cycle and mitosis in barley root cells remains limited. Here, we analyzed barley root growth across a temperature gradient from 15°C to 37°C, encompassing ambient, thermomorphogenic, and HS conditions. Root growth was stimulated by moderately elevated temperatures but arrested at approximately 35°C. HS-changed nuclear architecture parameters, including expanded nuclear area and altered circularity. Although HS led to a temporary mitosis arrest, we demonstrated that DNA replication and mitotic activity were efficiently reinitiated upon recovery at a lower temperature. Finally, we showed that moderately higher temperatures speed up mitosis. Notably, anaphase was the least affected compared to other mitotic phases. In summary, we show that germinating barley plantlets sustain active growth at high speed to temperatures above 30°C and that HS blocks cell cycle around the two critical cell cycle stages – S phase and mitosis in barley. These observations expand the knowledge of barley root growth under high temperatures and will help develop HS-resilient cereals.

## 1 Introduction

Roots have manifold and unique functions to anchor plants in a substrate, acquire water and minerals to support plant growth, and interact with the soil microbial communities (Ryan et al., 2016; Hodge et al., 2009). A well-developed root system is essential for plants to prosper and provide a high yield. Roots also represent one of the fastest-growing plant organs, which is achieved by an active cell division in the root meristematic zone and a combination of cell division and elongation in the root elongation and differentiation zones (Gao et al., 2014; Dolan and Davies, 2004; Ivanov, 1997). The actively dividing meristematic cells undergo a regular cell cycle with G0/G1, S, G2, and mitotic phases. In some plants, cells in the differentiated zone also integrate the endoreduplication cycle, where the G2 phase is followed by another S-phase (Desvoyes et al., 2021). Understanding the mechanisms of root growth and their responses to the environment is essential for plant breeding and the development of stress-resilient crops (Kohli et al., 2022; Lombardi et al., 2021).

Cultivated barley (*Hordeum vulgare* L.) is an ancient crop used mainly for animal feed, in the malting industry, and regionally as human food (Jiang et al., 2025). The diploid nature, large genetic resources, assembled genomes, and accessibility for genome engineering make barley an excellent temperate cereal model for basic and applied research (Hansson et al., 2024; Dawson et al., 2015). Like other cereals, barley has a fibrous root system with two main root types (Maqbool et al., 2022; Liu et al., 2020). Typically, four to eight seminal roots emerge directly from the embryo during germination and early seedling development. Later, nodal (crown) roots proliferate from the nodes at the bases of the stems (Reid, 1985). Both root types differ in anatomy, transcriptomic and proteomic profiles, and nutrient uptake capacity (Liu et al., 2020; Loades et al., 2015). Successful establishment of seminal roots is essential for the initial growth phases, where the germinating plant has to switch from the heterotrophic embryonic phase to the phototrophic somatic phase. The genetic regulatory network that controls root growth responds to the biotic and abiotic factors, which provides a necessary balance between tissue proliferation and protection (Loades et al., 2015).

Temperature is an important factor influencing plant growth in a complex manner. Under warmer-than-normal temperatures, plants undergo thermomorphogenesis (Casal and Balasubramanian, 2019). This adaptive process includes a suite of epigenetic, physiological, and morphological changes to mitigate the adverse effects of elevated temperature. With further increasing temperature, plants may experience heat stress (HS), i.e., temperatures above a threshold level for normal cellular functions, sufficient to cause irreversible damage (Jagadish et al., 2021; Wahid et al., 2007). This may lead to the damage of organs, breakage of the cell homeostasis and photosynthetic activity, as well as massive changes in the organization of the cell nuclei, led by heterochromatin decondensation resulting into the enlargement of nuclei and changes of their shape and morphology, including reorganization of the nuclear lamina and envelope, nucleolus, cytoskeleton and microtubular network (Attar et al., 2024; Lee et al., 2024; Sun et al., 2020; Kaiserli et al., 2018; Müller et al., 2007). Under the natural conditions, the risk of HS episodes increases due to the ongoing climate change, which is associated with the extreme weather conditions in terms of frequency and amplitude. Therefore, great attention is being paid to the effects of HS and associated drought stress on crop production in terms of yield quantity and quality. The current estimates suggest HS-induced yield losses for various crops from 30-90% (Lai et al., 2025; Becker et al., 2023; Bartosiewicz and Jadczyszyn, 2021; Fahad et al., 2017). The rising global temperatures are also becoming a critical constraint for cereal production, particularly in the temperate to semi-arid regions (Bvenura and Kambizi, 2022). Barley might be among the most severely affected crops as it grows under a cooler climate than other cereals (Siddique et al., 2025; Zenda et al., 2022). The effects of HS on barley growth were assessed at both molecular and phenotypic levels. Several studies have analyzed transcriptional and proteomic responses of various barley cultivars (Mishra et al., 2024; Cantalapiedra et al., 2017; Ashoub et al., 2015; Mangelsen et al., 2011). The phenotypic effects of HS have been analyzed primarily during the inflorescence development and grain filling and support the hypothesis that the HS exposure during the reproductive development leads to reduced fertility and yield losses (Horváth et al., 2024; Callens et al., 2023; Mahalingam and Bregitzer, 2019; Gous et al., 2016).

However, relatively little is known about how the HS affects the growth of barley plants shortly after germination and during the juvenile stage. Therefore, we analyzed the dynamics of barley root growth under different temperature regimes, estimated cell cycle dynamics, duration of mitosis, and various nuclear morphology-related traits under the control and HS conditions. Our data indicate that barley responds quickly and dynamically to the HS stimuli by avoiding mitosis and halting cells before the S-phase of the cell cycle. Surprisingly, heat-stressed barley roots can restart their cell cycle soon after returning to the ambient temperatures.

## 2 Materials and methods

### 2.1 Plant materials and growth conditions

Two-row spring-type *H. vulgare* L. cv. Golden Promise (GP) was used in this study. The EYFP-H2B mCHERRY-TUA3 double fluorescent marker line (FML) in GP background was created by crossing single FMLs of *ZmUBI1:EYFP-HvH2B:T35S* (*H2B*; HORVU.MOREX.r3.0Hr1G006420) and *ZmUBI1:mCHERRY-TUA3:TNos* (*TUA3*; HORVU.MOREX.r3.4HG0338800) using CDS sequences amplified from GP alleles as described (Kaduchová et al., 2023b).

**Figure 1:**
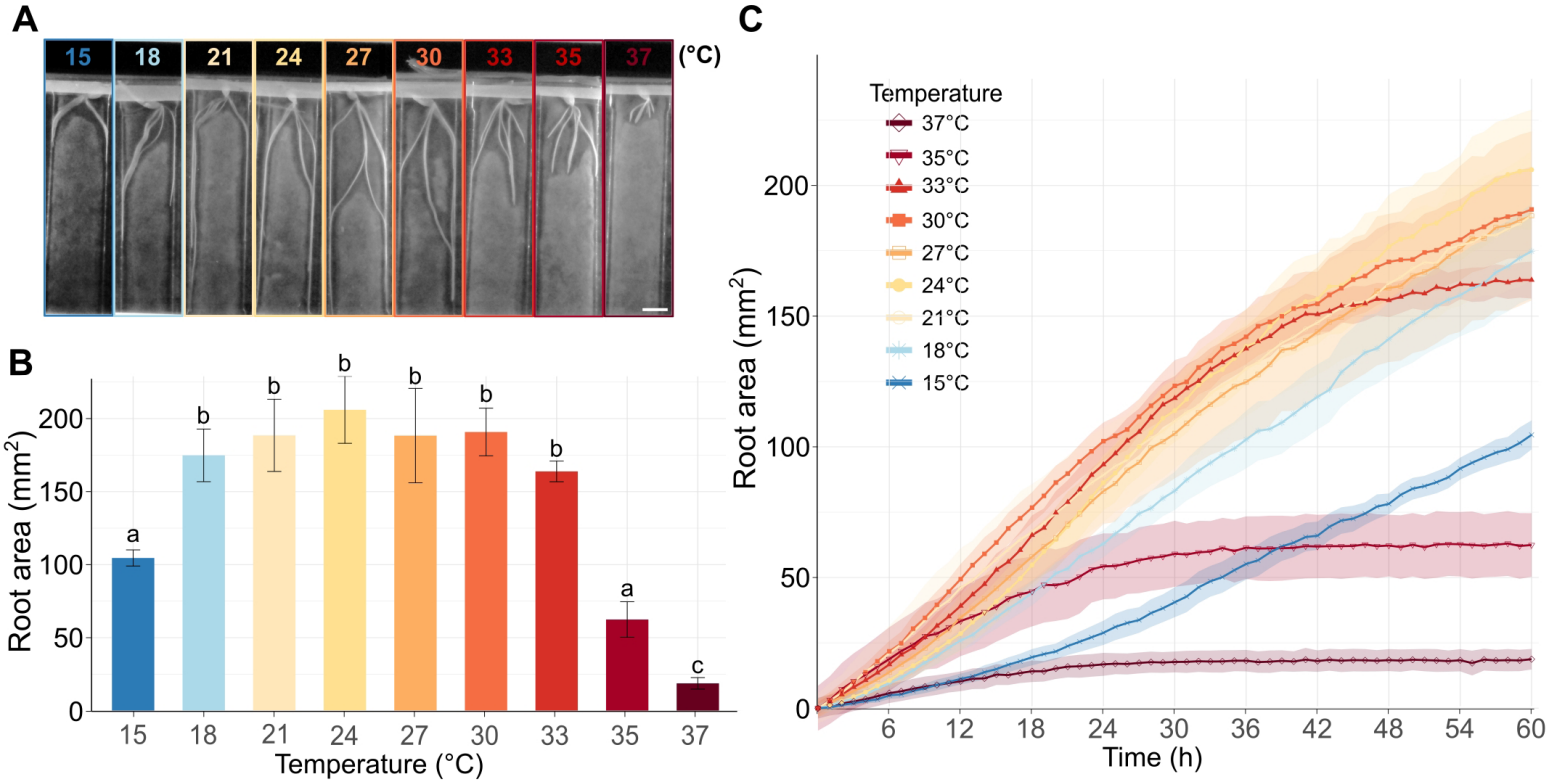
Barley root growth in response to increasing temperatures. **(A)** Representative barley seedlings grown for 60 h at the specified temperatures in the RaPiD chamber and photographed in the infrared mode. Scale bar = 1 cm. **(B)** The barplot shows a total mean root area (mm^2^) for roots of plants grown for 60 h at the given temperature, as described in **(A)**. The letters above bars represent significantly different groups at P < 0.05 in the Kruskal-Wallis test with Dunn’s *post-hoc* test and Benjamini–Hochberg correction. **(C)** Total root area (y-axis) at different time points (x-axis) and temperatures. The measurements were taken in 1 h intervals. **(B, C)** Error bars and shadowed error bars represent standard errors of means among four individual plants in each experimental time point.

All plant HS treatments are graphically summarized in **Supplementary Figure 1**. All experiments were performed in the dark as only plantlets lacking green parts were used. Seeds of both wild-type GP, and EYFP-H2B mCHERRY-TUA3 double FMLs were soaked in tap water on a wet filter paper, stratified in dark at 4°C for 48 h, and transferred for 12 h to 21°C for imbibition (to secure that only actively growing seeds of the same age will be used; exception was made for the *in vivo* mitosis duration experiment where only narrow range of treatment temperatures was used). For live root growth assays, imbibited wild-type GP seeds were mounted into 12 × 12 cm square Petri dishes, separated by plastic spacers, and covered by wet filter paper. The remaining space of the Petri dishes was filled with a moist soil substrate (Florcom). The dishes were sealed by Parafilm and placed into the RaPiD-chamber (Kashkan et al., 2022) in vertical orientation. To ensure the desired temperature (15, 18, 21, 24, 27, 30, 33, 35 and 37°C), the RaPiD-chamber was kept in the cooled incubator (VWR Collection) and the Petri dish was imaged in the dark mode at 1-hour intervals over 65-hour period, starting by an initial 5-hour temperature acclimatization (**Figure 1**; **Supplementary Video 1**). For mitotic index analysis, imbibited seeds of EYFP-H2B mCHERRY-TUA3 FMLs on Petri dishes were transferred to 15, 18, 21, 24, 27, 30, 33, 35, or 37°C for 24 h, and individual germinated plants were used for microscopy. For the analysis of mitosis duration *in planta*, seeds of EYFP-H2B mCHERRY-TUA3 were germinated directly at 18, 21, or 24°C for 48 h in the dark due to slight differences in treatment temperatures, and to overcome a potential undesired influence of the transfer for the *in planta* microscopy. For nuclear DNA content measurements and EdU pulse labeling, wild-type GP germinated seeds were placed on Petri dishes and then moved to 37°C in the dark for the desired time (HS regimes) or back to 21°C in the dark for recovery.

### 2.2 Live root growth assays and semi-automated phenotyping of barley roots

The segmentation of time-lapse captured root growth on varying temperatures was performed by a RootPainter software, which provided an intuitive graphical user interface and human-in-the-loop approach for training a U-net convolutional neural network (Smith et al., 2022). A dataset of 116 photos was used to train the model by manual annotation to distinguish root from background pixels using the Google Colab (Google, 2025) computing resources. The model was further tuned for each temperature dataset to achieve the best segmentation accuracy. Segmented images were converted to RhizoVision format to be skeletonized and evaluated in RhizoVision Explorer v2.0.3 (Seethepalli and York, 2020; Seethepalli et al., 2021). Regions of interest were manually defined around each seedling, and datasets were analyzed in batches with semi-default settings (Whole root analysis mode, Convert pixels to physical units = 308 DPI, Keep largest component, Edge smoothing threshold = 2). The pixels were recalculated to DPI in Fiji software (Schindelin et al., 2012). Growth gain of the network area (mm^2^) evaluated in RhizoVision was plotted as an average for the roots of four individual seedlings in R software using ggplot2 (Posit team, 2025). Time-lapse videos of the root growth were generated using Fiji software (Schindelin et al., 2012) and merged in Clipchamp software (Microsoft).

### 2.3 Nuclear DNA content measurements and estimation of cell cycle dynamics

Nuclear DNA content was measured immediately after the control and HS treatments as specified in the section ‘growth conditions’. Samples were prepared by dissecting and pooling 1 mm long tips of five roots, homogenization by manual chopping with a razor blade in LB01 homogenization buffer containing 0.2 μg/ml 4’,6-diamidine-2-fenylindole (DAPI) as described (Doležel, 2007). The proportions of nuclei with different DNA content were analyzed on Partec PAS I flow cytometer (Sysmex, Kobe, Japan), where RN1 was considered as 2C, RN2 as 4C, and RN3 as 8C nuclei. The S-phase nuclei cannot be unambiguously distinguished from the nuclei populations forming the bases of the 2C and 4C peaks using this method, and therefore were not quantified.

### 2.4 *In planta* confocal laser scanning microscopy

All microscopic images were acquired using a Leica TCS SP8 STED3X confocal microscope (Leica Microsystems, Wetzlar, Germany), equipped with a white excitation laser, an HC PL APO CS2 20x/0,75 NA DRY objective, hybrid detectors (HyD), and the Leica Application Suite X (LAS-X) software version 5.3.0 with the Navigator module (Leica, Buffalo Grove, IL, USA). The fluorophore excitation was performed subsequently using the following excitation laser wavelengths: 514 nm for EYFP, 587 nm for mCHERRY, 405 nm for DAPI, and 499 nm for Alexa488. The emission signals were detected using HyD detectors (519–584 nm EYFP, 592–784 nm mCHERRY, 410–494 nm DAPI, and 504–690 nm for Alexa488).

For the mitotic index analysis, microscopic Z-stack images of the top rhizodermal cell layer were taken with 2.5 × optical zoom and covered two camera view fields (233 × 233 µm), distanced (233 µm) from a root quiescent center using the Leica LasX Navigator module. Numbers of mitotic and interphase cells were counted manually using the CellCount package in ImageJ Fiji version 1.53c (Schindelin et al., 2012). The same images were used to analyze average meristematic root cell length, where the lengths of individual cells within one cell file at each treatment temperature were measured manually using ImageJ Fiji. Statistical analysis was done using RStudio version 5.3.0 (Posit team, 2025).

To analyze the duration of mitotic stages, germinated seeds were mounted in tap water into a tempered EasyClick microscopy holder (Kaduchová et al., 2023a). Z-stack images of 20-40 μm width were captured in 45 or 60 second intervals for at least 45 minutes based on the root mounting quality and the total length of the scanning. Duration of individual mitotic stages was calculated manually using the Leica Application Suite X (LAS-X) software version 5.3.0. Statistical analysis was done using RStudio version 5.3.0 (Posit team, 2025).

### 2.5 EdU pulse labeling

The EdU pulse labeling was performed using a customized protocol. Treated seedlings were incubated for 30 min in 20 µM EdU (Click-iT™ EdU Alexa Fluor™ 488 Flow Cytometry Assay Kit, ThermoFisher Scientific/Invitrogen, Waltham, MA, USA) in ddH_2_O at 21°C (control), or at 37°C for 3 h HS samples. Afterward, roots were fixed in 4% (v/v) formaldehyde in Tris buffer (10 mM Tris, 10 mM Na_2_EDTA, 100 mM NaCl, 0.1% Triton X-100, pH 7.5) for 45 min at 4°C, and washed three times in Tris buffer at 4°C (Doležel, 2007). Next, roots were treated with a 0.5 ml Click-iT reaction cocktail (Click-iT™ EdU Alexa Fluor™ 488 Flow Cytometry Assay Kit), prepared according to the manufacturer’s instructions, and incubated for 10 min in a vacuum, followed by their incubation in the dark for 45 min at room temperature. After labeling, roots were washed three times for 5 min in phosphate-buffered saline (PBS; 10 mM Na_2_HPO_4_, 2 mM KH_2_PO_4_, 137 mM NaCl, 2.7 mM KCl, pH 7.4) at room temperature. Subsequently, nuclei were stained by incubating roots in DAPI (100 µg/ml; 0.1% Triton X-100) with and without vacuum (15 min each, then washed 3 times in PBS buffer). Microscopic pictures of stained roots were acquired using a Leica TCS SP8 STED3X confocal microscope (Leica Microsystems, Wetzlar, Germany) as described for the counting of mitotic index. For the quantification, maximal projections of Z-stacks (9-12 µm total width) comprising ten layers were analyzed using ImageJ Fiji (Schindelin et al., 2012) by defining the fluorescent signal thresholds for both Alexa488 and DAPI in the centered defined ROIs (700 x 200 µm) with manual adjustments. Five figures were analyzed per replicate.

## 3 Results

### 3.1 Increasing temperature accelerates root growth speed

To explore how increasing temperature influences the dynamics of barley root growth, we set up a time-lapse analysis of the roots of germinated barley seeds exposed to temperatures from 15°C to 37°C. The initial experiments revealed that the applied temperature range affected barley seed germination (**Supplementary Figure 2**). The germination stopped or was significantly delayed at the highest or the lowest applied temperatures. To avoid the combined effects of HS on germination and post-germinative root growth, we germinated the seeds for 12 h at an identical temperature of 21°C. This ensured robust setup of our assays and comparability of root growth among different temperatures (**Supplementary Figure 1A**). The plants were photographed by the RaPiD-chamber (Kashkan et al., 2022), designed for time-lapse photo acquisition of plants grown on Petri dishes under controlled conditions. The root growth was monitored in one-hour intervals for up to 60 h (**Supplementary Video 1**). The image acquisition was followed by semi-automatized machine-learning and data post-processing using a customized published protocol (Callwood et al., 2025). As a proxy for the root growth, we calculated the gain of root biomass in mm^2^ for every temperature and time point (**Figures 1A–C**; **Supplementary Datasets 1, 2**). This parameter was preferred over the average root length because the roots often grew alongside each other at later time points, and could not be securely distinguished using automated data post-processing. At the lowest applied temperature (15°C), the roots grew slowly but continuously over the whole monitoring period (**Figure 1C**, **Supplementary Dataset 2**). The increasing temperature sped up the growth, and the roots showed similar gain in the growth area in the range from 18°C to 27°C, and the roots gained the highest biomass at 24°C (**Figures 1A–C**). At the temperature range from 30°C to 33°C, the growth was initially similar, or even faster, compared to the lower temperatures, but lasted only for about 42 h and then rapidly slowed down (**Figures 1B, C**). This trend was even more pronounced for the plants grown at 35°C, where the initially fast growth slowed down between 12 and 24 h and stalled for the remaining duration of the experiment. Finally, the roots of plants grown at 37°C showed minimal growth from 0 to 24 h and no growth at later time points (**Figures 1B, C**).

Overall, our data indicate that the increasing incubation temperature stimulates the root growth within a relatively broad range of temperatures from 18 to 30°C, with a long-term (60 h) growth maximum at 24°C for the tested cultivar Golden Promise. The temperatures of 35°C and higher led to a sharp decline in the seminal root growth.

### 3.2 Temperature-dependent root growth correlates with mitotic activity

**Figure 2:**
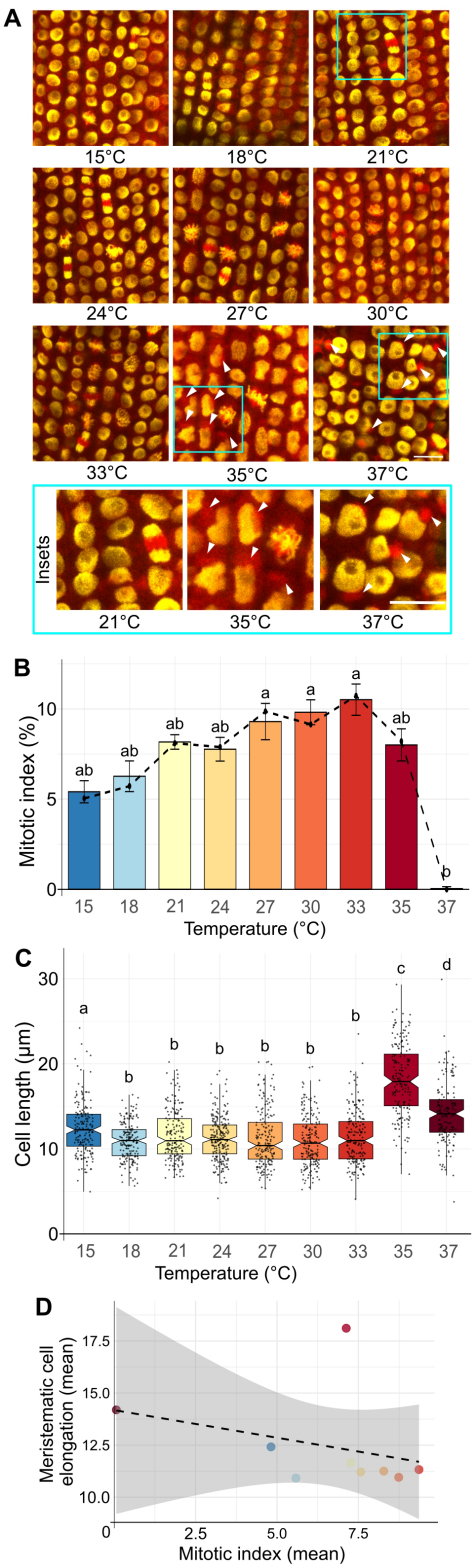
Effect of increasing temperature on the mitotic index frequency. **(A)** Representative images of EYFP-H2B (yellow), mCHERRY-TUA3 (red) fluorescent marker line in the rhizodermal cells of the meristematic zone at different temperatures. Cyan rectangles show enlarged sections for control (21°C) and HS temperatures with visibly collapsed microtubular network and enlarged nuclei of irregular shape. White arrows at 35 and 37°C indicate heat stress-induced collapse of the microtubular network into large cytoplasmic speckles. 300 to 650 cells were analyzed in 5 roots per time-point. Scale bar = 20 µm. **(B)** Frequencies of mitotic index in barley roots after 24 h at the given temperature. The dashed solid line connects median values for individual temperatures. Error bars represent the standard error of means among five individual plants analyzed per temperature. At least 1700 cells were examined for each treatment. The letters above bars represent significantly different groups at P < 0.05 in the Kruskal-Wallis test with Dunn’s *post-hoc* test. **(C)** Cell length in barley meristematic root cells. Notched boxplots show variation in cell length; the thick black line within the boxes marks the median, upper and lower boxes correspond to the first and third quartiles of the data, respectively, and the lower and upper whiskers mark 10 and 90 percent intervals, respectively. The black points represent individual measurements. n > 149 cells per sample. Bars with different letters above statistically significantly differ at P ≤ 0.001 in the Kruskal-Wallis test, followed by the Wilcoxon *post-hoc* test. **(D)** Correlation between the mitotic index (x-axis) and cell length (y-axis) over the temperature gradient from 15 to 37°C in barley root meristematic zone. Pearson correlation coefficient r = -0.317; P = 0.407, grey section represents 95% confidence interval (95% CI = [–0.810, 0.440]). Spearman correlation coefficient ρ = -0.450; P = 0.230.

To explore how increasing temperature influences barley root growth at the cellular level, we used EYFP-H2B mCHERRY-TUA3 FMLs that were designed for *in planta* visualization of chromatin and microtubule dynamics (Kaduchová et al., 2023b). The cold-stratified seeds were germinated for 12 h at 21°C for the emergence of the primary root, then incubated at a series of temperatures (15, 18, 21, 24, 27, 30, 33, 35, 37°C) for 24 h, and analyzed by microscopy (**Figures 2A, B**; **Supplementary Figure 1B**).

First, we analyzed mitotic index (MI) in the meristematic zone of barley roots. The MI steadily increased from 4.8% at 15°C to 9.4% at 33°C (mean values: 4.8, 5.6, 7.3, 7.6, 8.3, 8.7, 9.4% for 15, 18, 21, 24, 27, 30, and 33°C, respectively; **Supplementary Dataset 3**). The MI reached 7.1% at 35°C, indicating a decrease, followed by a sharp reduction in MI to 0.045% at 37°C (**Figure 2B**). The remarkable absence of mitosis at 37°C corresponded with the observations from the RaPiD-chamber, indicating that this temperature is a severe HS for barley and leads to a growth arrest (**Figure 2B**). Accordingly, we found temperature-dependent differences in the cell organization from 33 to 37°C (**Figure 2A**; insets in cyan frame). At 35 and 37°C heat stress, the microtubule network (mCherry-TUA3) significantly redistributed from filamentous structure to clusters at the proximity of nuclear membrane (33% and 100%, respectively, versus 0% at 21°C; **Figure 2A**; **Supplementary Figures 3A, B**). Consequently, the architecture of the cells in the root apical meristem (RAM) was also impaired. The nuclei had a larger area, with distinctly visible nucleolar cavities (round structures within nuclei lacking EYFP signal; **Figure 2A**, 37°C). The 37°C heat stress also led to irregular pattern of cells within the cell files, and even missing cell as indicated by the positons without EYFP-H2B signals (**Supplementary Figure 3A**, white arrows). To some extent, nuclear shape and chromatin condensation alterations were detectable already at 35°C (**Figure 2A**, white arrows). Although some cells already displayed destruction of the microtubular network at this temperature, they still had a high 7.1% mitotic index. This also agrees with the initial high barley root growth kinetics at 35°C (**Figure 1C**). Moreover, we wanted to investigate if HS also affects the lengths of the cells within the root meristematic zone. We analyzed the lengths of the cells in samples grown at 15 to 37°C temperature gradient and found that at 35 and 37°C, the cells underwent an elongation (means 18.1 and 14.2 µm) compared to lower (non HS) temperatures (**Figure 2C**; **Supplementary Dataset 4**). Further, we observed increased cell elongation in roots grown at 15°C (mean 12.4 µm compared to 10.9 to 11.7 µm for 18 to 30°C). We assume that roots grown at sub-optimal temperatures (e.g., close to cold or heat stress) tend to prolongmore by the cell elongation and not by mitosis (i.e., negative correlation between meristematic root cell and mitotic index) (**Figure 2D**).

Hence, we showed that the HS-induced inhibition of root mitotic activity becomes prominent from 35°C, including the collapse of the microtubule network and changes in cellular and nuclear shape, area, and cell length.

### 3.3 HS-induced suppression of mitosis is reversible

**Figure 3:**
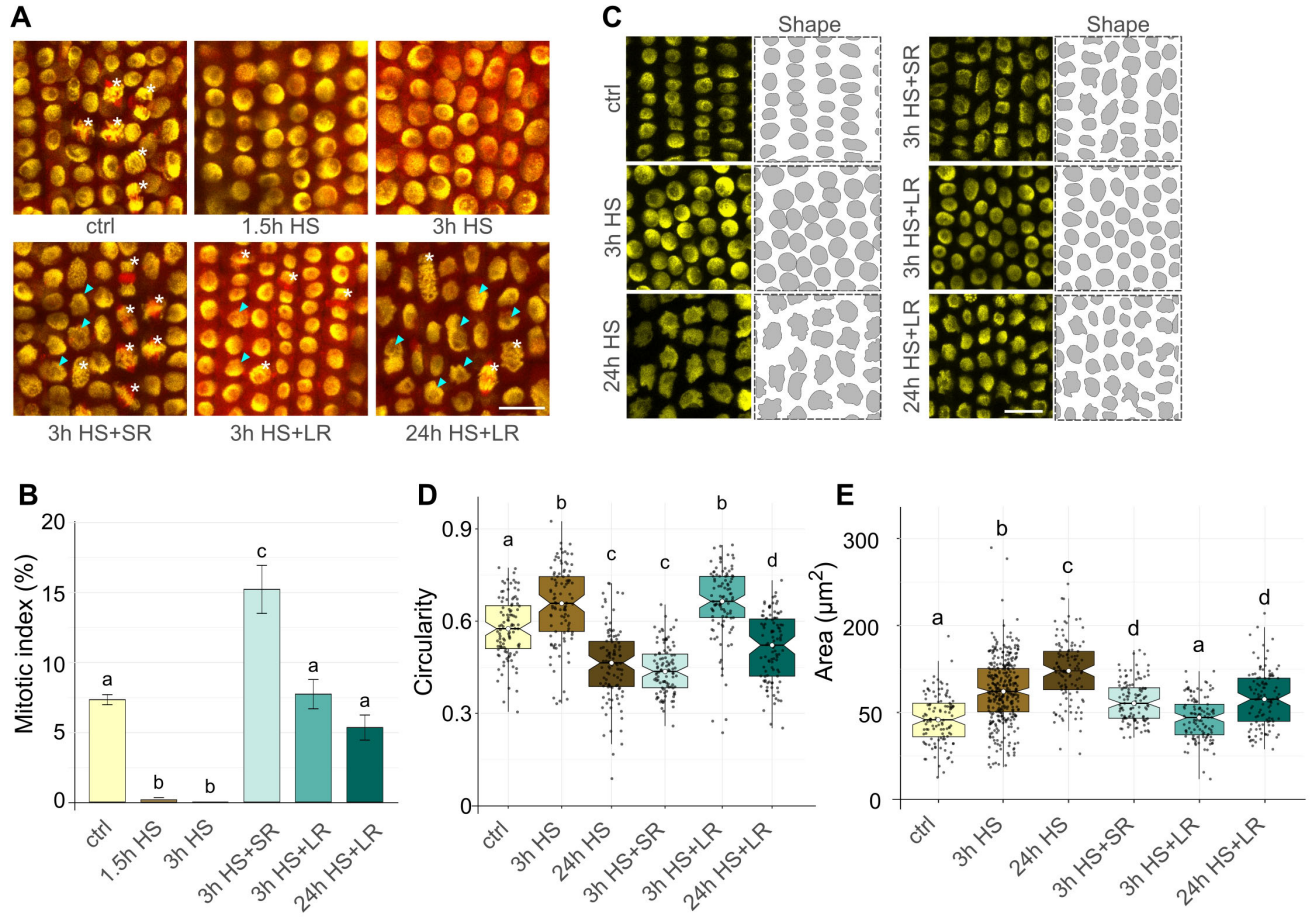
Heat stress (HS) recovery affects mitotic division and nuclear morphology. **(A)** Representative EYFP-H2B (yellow) mCHERRY-TUA3 (red) marker line rhizodermal cells at 21°C control (ctrl), 37°C HS, and 3h short (SR) and 24 h long (LR) recovery at 21°C regimes. Cells at different stages of mitosis (white asterisk) and enlarged nuclei with irregular shape (cyan arrows) are indicated. Scale bar = 20 µm. **(B)** Bar plot representing a mitotic index at regimes specified in **(A)**. Error bars represent the standard error of the means among five individual plants per temperature, and at least 1800 cells were analyzed per treatment. Bars with different letters above statistically significantly differ at P ≤ 0.05 in the Kruskal-Wallis test, followed by a Wilcoxon *post-hoc* test. **(C)** Shape segmentation (right) of EYFP-H2B labeled nuclei (left) in ctrl, 3 h, and 24 h HS treatments and after short (SR) and long (LR) recovery at 21°C. Scale bar = 20 µm. **(D)** Notched boxplots show nuclear circularity after treatments described in **(C)**; the thick black line within the boxes marks the median, the upper and lower boxes correspond to the first and third quartiles of the data, respectively, and the lower and upper whiskers mark 10 and 90 percent intervals, respectively. The black points represent individual measurements. n = 102 nuclei per sample. Bars with different letters above statistically significantly differ at P ≤ 0.001 in the Kruskal-Wallis test, followed by the Wilcoxon *post-hoc* test. **(E)** Notched boxplots show change in nuclear area (µm^2^) after treatments specified in **(C)**. The boxplots and statistical test are as described in **(D)**. n = 113 nuclei per sample.

The root phenotypes and microscopy-based analysis suggested that the acute HS of 37°C strongly suppressed cell divisions and RAM proliferation (**Figures 1**, **2**). However, under natural conditions, HS frequently diminishes after some time, and the surrounding temperature gradually decreases from the thermomorphogenic to the ambient level. To assess the responses of barley meristems to the post-stress situations, we analyzed root growth under various simulated recovery conditions (**Figure 3**; **Supplementary Figure 1C**).

The EYFP-H2B mCHERRY-TUA3 FML seedlings were exposed to 37°C HS for 1.5, 3, or 24 h, transferred to the control (21°C) conditions for a short 3 h (SR) or a long 24 h (LR) recovery period, followed by analysis of mitosis. The 1.5 h and 3 h HS almost and/or entirely abolished mitosis in RAM (mitotic indexes 0.16% and 0%, respectively; **Figures 3A, B**; **Supplementary Dataset 3**). However, we observed MI of 15.1% after 3 h HS + SR, representing a doubling of mitotic activity relative to the control conditions with the mitotic index of 7.27% (**Figure 3B**). After 3 h HS + LR, we found 7.66% mitotic cells, corresponding to the values observed for control conditions. This interesting observation suggests that the 3 h HS stops some barley cells at a specific cell cycle stage and leads to their synchronized progression throughout the post-HS cell cycle, including mitosis. However, after 24 h LR, the synchronization diminished, and the cells divided at a regular frequency. Moreover, even the RAMs exposed to a severe 24 h 37°C HS followed by 24 h LR showed a mitotic index of 5.29%, indicating almost complete recovery of the mitotic activity (**Figures 3A, B**).

Next, we analyzed the effects of the 3 h and 24 h HS on barley nuclear morphology by quantifying nuclear circularity (0 = not circular; 1 = perfectly round) and area (**Figures 3C-E**; **Supplementary Dataset 5**), as these parameters may serve as broad indicators of the overall HS-induced changes of nuclear architecture. Surprisingly, the circularity of nuclei varied profoundly after the HS exposure. After 3 h HS, the nuclei became significantly rounder compared to the control conditions (circularity medians 0.65 and 0.58). However, 24 h HS had an adverse effect and significantly decreased circularity (median 0.46) compared to both control and 3 h HS treatments (**Figures 3C, D**). After recovery, we observed no significant changes after 3 h (SR) compared to 24 h HS (median 0.44), but it leveled with 3 h HS after 24 h of LR (median 0.66). For 24 h HS + LR, circularity value (median 0.52) was between the ctrl and 3 h HS medians.

On the contrary, nuclear area increased significantly with a prolonged influence of HS for both 3 h and 24 h treatments (medians 62.13 and 75.57 µm^2^) and also 3 h HS + SR and 24 h HS + LR (medians 55.25 and 57.65 µm^2^) compared to control (median 48.96 µm^2^), which was comparable with values for 3 h HS + LR (median 47.11 µm^2^) (**Figure 3E**; **Supplementary Dataset 6**).

These observations indicate that the HS-induced suppression of mitotic activity of RAM cells leads to an increase in mitotic divisions within the first hours after the return to normal conditions and that HS alters nuclear morphology and size.

### 3.4 Heat stress affects the cell cycle in RAM

**Figure 4:**
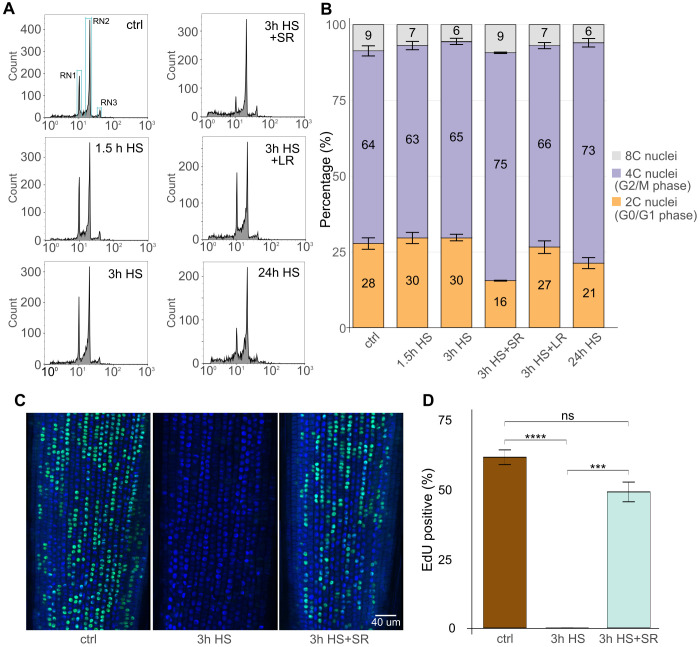
Heat stress (HS) restricts cell cycle progression in the G2 phase and replication. **(A)** Flow cytometry histograms in logarithmic display representing 2C/G1 (RN1), 4C/G2 (RN2), and 8C/endoreduplicated (RN3) nuclei in control (ctrl) and HS-treated and short 3 h or 24 h long recovered (SR and LR, respectively) samples. The number of particles in each defined region was retrieved for each sample for quantitative analysis. **(B)** Stacked bar plot representing percentage of 2C/G1 (orange), 4C/G2 (purple), and C8/endoreduplicated (grey) nuclei as defined in **(A)**. Three biological replicates per treatment were measured, and their mean values were used. Standard error bars of the mean are shown for each bar segment. Statistics were done using the Kruskal-Wallis test and the Wilcoxon *post-hoc* test. P values for 2C/4C/8C for ctrl vs. treatments as follows (P = 0.66/1.00/0.19; 0.38/1.00/0.08; 0.08/0.08/0.66; 1.00/0.38/0.38; 0.08/0.08/0.08). **(C)** EdU labelling of the representative roots of ctrl, 3h HS, and/or in combination with short recovery. DAPI-stained nuclei (blue), EdU-positive nuclei (green). Scale bar = 40 µm. **(D)** Percentage of EdU-positive nuclei in individual samples. Error bars represent the standard error of the means among five individual plants and at least 2500 cells per treatment. Statistical analysis was done using the Kruskal-Wallis statistical test and Dunn’s *post-hoc* test; P ≤ 0.001. *** → p < 0.001, **** → p < 0.0001.

The increase in mitotic divisions following 3 h HS + SR suggested possible cell cycle synchronization by HS in barley. To find at which stage the cells might be stalled during HS, we explored cell cycle dynamics upon HS and various recovery regimes. Germinated barley plants were exposed to 1.5 h HS without recovery, 3 h HS without and with SR and LR, and 24 h HS, followed by the isolation of nuclei from the RAMs and their flow cytometry analysis. We estimated proportions of nuclei with different C-values serving as a proxy for the G0/G1 (2C, RN1), G2 (4C, RN2), and endoreduplicated (8C, RN3) nuclei (**Figures 4A, B**; **Supplementary Figures 1C, D**; **Supplementary Dataset 7**). The S-phase nuclei were not quantified because this methodology does not provide their reliable quantification relative to the statistically distributed signals of the G1 and G2 nuclei, which also overlap with the S-phase region. Under control 21°C conditions, barley RAMs contained 28% 2C, 64% 4C, and 9% 8C nuclei. The profile remained similar after 1.5 h and 3 h of 37°C HS with 30% 2C nuclei, 63 or 65% 4C nuclei, and 7 or 6% 8C nuclei, respectively. After 3 h of recovery (3 h HS + SR), there was a striking reduction in the proportion of 2C nuclei (16%) and an increase in the frequency of 4C nuclei (75%). For the 3 h HS + LR, the proportions of individual C-value populations returned to a control-like situation (27% 2C, 66% 4C, and 7% 8C). Simple 24 h HS treatment led to a partial reduction in 2C (21%) and a gain in 4C (73%) nuclei.

To reliably assess the effect of HS on the S-phase, we took advantage of the Click-iT EdU *in vivo* labeling of the replicating cells. The EdU was applied as a 30-minute pulse followed by the fixation of the sample. The control 21°C samples showed 61% EdU-positive nuclei organized in individual cell files (**Figures 4C, D**; **Supplementary Dataset 8**. On the contrary, there were no EdU-positive nuclei (0%) after 3h HS treatment, indicating a complete block of DNA replication. Upon 3 h recovery (3 h HS + SR), the replication restarted as indicated by 49% EdU-positive cells, but was lower than in control conditions.

These data show that a short 3 h HS already affects the cell cycle in RAM and that the regular cell cycle is re-established during recovery.

### 3.5 Moderately increased temperatures speed up barley mitosis

Next, we tested whether the temperature-stimulated root growth also affects mitosis duration. Two-day-old seedlings of the EYFP-H2B mCHERRY-TUA3 FMLs were mounted into the EasyClick microscopy holder (Kaduchová et al., 2023a), tempered to 18, 21, and 24°C (**Supplementary Figure 1E**), and the progression of mitosis was monitored from prometaphase to cytokinesis as described (Kaduchová et al., 2023b).

**Figure 5:**
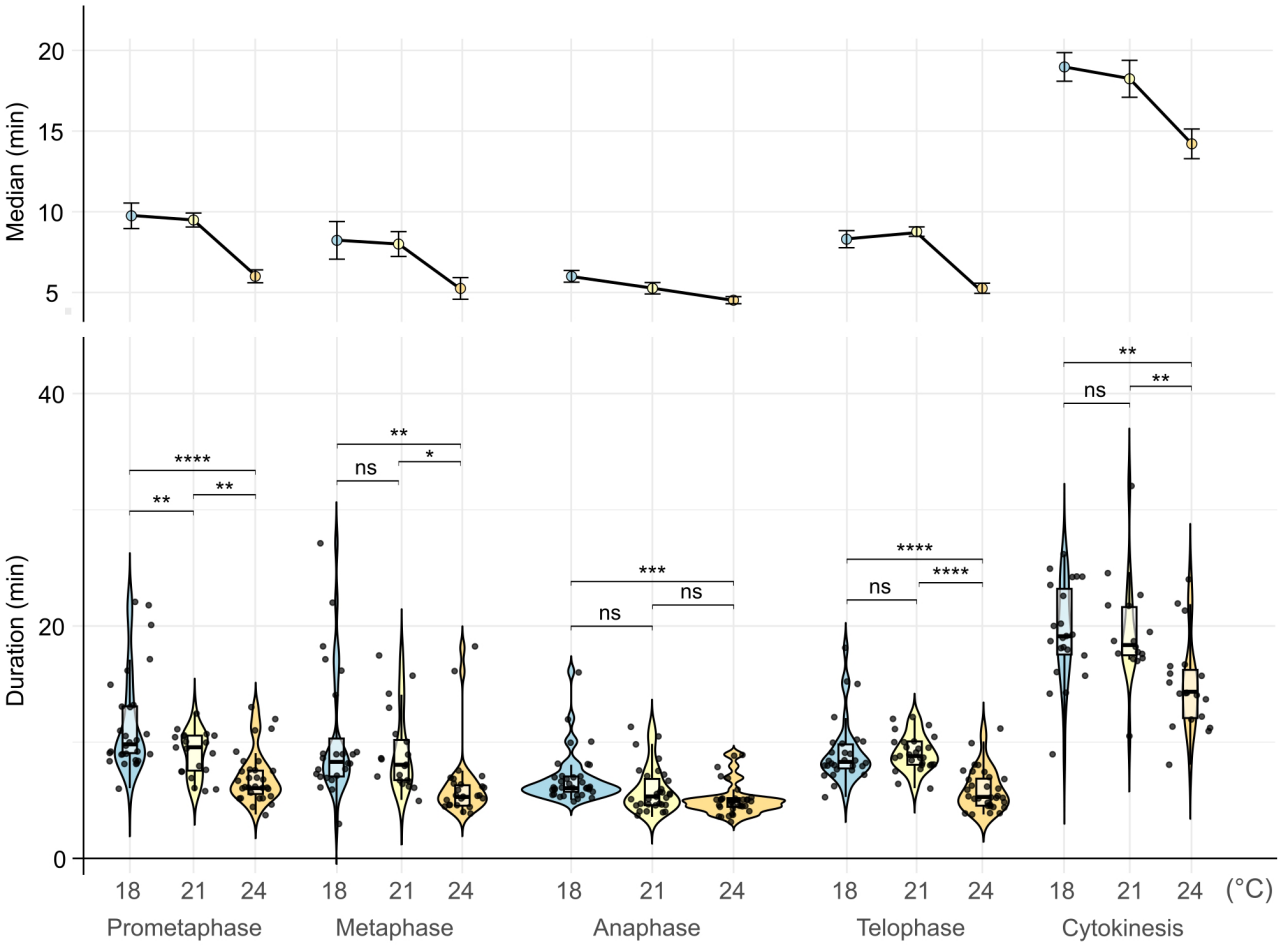
Duration of mitotic stages at different temperatures. Violin plots represent the distribution of mitosis stage durations. Boxes within violin plots correspond to the first and third quartiles of the data, respectively; the thick black line within the boxes marks the median; black points represent individual measurements. The lower and the upper whiskers mark 10 and 90 percent intervals, respectively. Statistical analysis was done using a one-way ANOVA statistical test, P ≤ 0.05, factor 1 = mitotic phase; n > 16 cells per stage. * → p < 0.05, ** → p < 0.01, *** → p < 0.001, **** → p < 0.0001.

The duration of most mitotic phases was not significantly changed between 18°C and 21°C, however the progression became substantially faster for all stages at 24°C except of the anaphase (median duration times at 18/21/24°C: prometaphase: 9.75/9.5/6 min, metaphase: 8.25/8.00/5.25 min, anaphase: 6/5.25/4.5 min, telophase: 8.25/8.75/5.25 min, and cytokinesis: 19/18.25/14.25 min, respectively) (**Figure 5**; **Supplementary Dataset 9**). This suggests that increasing temperatures can significantly accelerate the progression of most of the mitotic phases except for the anaphase. On the contrary, anaphase is the shortest phase of mitosis, and its duration is most likely already defined by a relatively constant speed of the kinetochore microtubule depolymerization in eukaryotes (Zhai et al., 1995; Zwetsloot et al., 2018; Maddox et al., 2003). Moreover, the distribution of individual measurements was narrower at higher temperatures, indicating a more stringent synchronization of the mitotic cycle. This agrees with general observations of the cell cycle and mitosis dynamics (Araujo et al., 2016; Santos et al., 2012). Finally, we also estimated the total median duration of mitosis from prometaphase to telophase as 32.25 min at 18°C, 31.5 min at 21°C, and 21 min at 24°C or 43, 41, and 30 min if cytokinesis is included, respectively.

In summary, these observations suggest that increasing non-HS temperatures accelerate mitotic division and cytokinesis.

## 4 Discussion

Heat is a prominent abiotic factor with adverse effects on plants. At the level of HS, it negatively influences the architecture of plant roots, leads to reduced nutrient and water uptake, and results in altered plant growth and decreased harvest yields (Mahalingam and Bregitzer, 2019; Fahad et al., 2017; Wahid et al., 2007). Here, we analyzed the effects of temperature gradient from 15 to 37°C on barley seedling root growth and their nuclear parameters.

Live analysis of barley root growth revealed temperature-dependent effects. At low temperature (15°C), the root growth was slow but continuous, though it sped up with increasing temperature, and was comparable for a range of temperatures from 18 to 33°C. With the higher temperature (35°C), the growth was initially high but stopped within the first 24 h of the treatment. Poor root growth followed by its stop was characteristic for 37°C. In Arabidopsis, increasing temperature is efficiently sensed already in the primary roots, leading to their growth promotion, cell elongation, and faster lateral root emergence (Ai et al., 2023; Bellstaedt et al., 2019; Feraru et al., 2019; Wang et al., 2016). The thermomorphogenic temperatures significantly accelerated root growth also in garden pea (Gladish and Rost, 1993). We were also able to confirm these observations in barley. Since our experiments were conducted on seedlings and tissues without green pigments in the dark, we speculate that roots of young barley seedlings grown in HS conditions might temporarily cope with the negative effects using the energy and other resources from the seed endosperm (Yan et al., 2014). However, dealing with the impact of HS might lead to a rapid exhaustion of energy and result in the root growth arrest.

Previous studies showed that thermomorphogenic temperatures positively influence mitosis and lead to higher division rates due to acceleration of cellular metabolism and signaling (Jacob et al., 2025), faster microtubule polymerization and depolymerization (Fuseler, 1975), and relaxed cell cycle checkpoint (Jang et al., 2005). Temperature-dependent adjustment of mitosis was previously shown also for barley (Brown, 1951). We observed dynamic responses of mitosis to different temperatures with some novel insights. At the ambient and thermomorphogenic range (up to 33°C), we found an increasing MI that was followed by a sharp reduction in MI from 35 to 37°C. After 1.5 h at 37°C, there were only a few mitotically active cells, and no division was observed after 3 h at 37°C. A similar trend was also visible in rye, with only very few mitoses occurring after 4 h of 40°C HS (Neves et al., 2020). Nevertheless, there was a small but measurable growth of the roots with the first 24 h of 37°C treatment in barley (**Figure 1C**). It has been demonstrated that roots grow by a combination of active mitotic cell division and cell elongation (Ai et al., 2023). Therefore, we estimated cell elongation in the root meristematic zone of barley plants grown at different temperatures. Meristematic cells had a similar length across the range of temperatures, but there was a remarkable extension of meristematic cell length for plants grown at 35°C. The correlation plot revealed a negative relationship between mitotic activity and cell length parameters, i.e., the cells were shorter in roots with actively dividing cells and longer in the roots with suppressed mitosis. The striking absence of cell elongation at 37°C could be caused by the severity of such HS treatment for barley. This is consistent with the observation that HS frequently leads to cell death in plants as observed in Arabidopsis or maize roots (Wang et al., 2025; González-García et al., 2023). Also, it has to be noted that our estimation was limited to the root meristematic zone. The cell elongation in response to HS might be more pronounced in the differentiated zone (Ai et al., 2023). This showed that the meristematic zone of barley roots grows primarily by active cell division under non-HS conditions but suppresses mitosis and, to a limited extent, leads to cell elongation in the meristematic zone. Interestingly, the HS-induced suppression of mitosis was conditional, and the mitotic activity was restored during recovery at ambient temperature. In fact, there were twice as many mitotic cells at 3 h of recovery compared to normal conditions, indicating that HS, to some extent, synchronizes the cell cycle. This is in agreement with findings in rye, where a short recovery period elevated mitotic activity (Neves et al., 2020).

HS led to microscopically detectable changes at the subcellular level. The microtubule network collapsed typically into a single large cytoplasmic nucleus-proximal domain at 35 and 37°C. Nevertheless, the microtubular activity was recovered and mitosis reinitiated during the recovery phase in barley, similarly to Arabidopsis and *N. tabacum* cells (Müller et al., 2007; Smertenko et al., 1997). In addition, HS-caused dynamic changes in nuclear morphology. The nuclear area generally increased after HS and required at least 24 h of recovery to return to control values. The HS also initially increased circularity of nuclei similar to Arabidopsis (Wang et al., 2023). After a longer exposure, the nuclear envelope developed grooves, and the shape became less circular. This resembles previous observations in rye roots, where HS resulted in nuclei with irregular shapes and micronuclei (Neves et al., 2020). HS-induced changes in nuclear morphology and intensity of chromatin modification signals were also observed in maize roots (Zhao et al., 2014). The changes in nuclear morphology are tightly connected with the overall stability of the nuclear lamina in plants (Wang et al., 2023). The striking loss of circularity after prolonged HS is probably caused by an abolition of cellular homeostasis, including microtubule network (Hoekstra et al., 2001). Such changes can also be cell-type dependent, as shown in Arabidopsis epidermal root cells, and the fast-dividing young cells are likely the most prone to disruption of the cytoskeleton and abortion of mitosis (Müller et al., 2007). However, the size of barley roots allowed microscopic analysis of only the epigermal cell layers.

Several of our observations suggested effects of HS on cell cycle progression. Even the short 1.5 h HS treatment suppressed mitosis. However, the whole process restarted during recovery, and we found a doubled frequency of dividing cells 3 h after HS. This strongly indicates that HS blocks the cell cycle before mitosis, likely at the G2-M checkpoint. This, to some extent, synchronizes the cells that enter mitosis at similar times during recovery and results in enrichment of mitotic cells. After 24 h recovery, the frequency of mitoses appeared normal, likely due to different cell cycle kinetics between individual cells. Furthermore, we argue for another less noticeable HS-induced cell cycle pause at the end of G1 or beginning of S-phase. The EdU labeling of replicating cells revealed a lack of signals after 3 h of HS, suggesting that no new G1 cells started replication during HS. After 3 h of recovery from 3 h HS, the proportion of G1 nuclei decreased to about one-half (16% versus 30% at 3 h HS; **Figure 4B**), and the number of replicating cells was similar to the control conditions (**Figure 4C**). Taken together, this suggested that the cells were halted also before replication during HS and rapidly progressed further during recovery. Hence, our data indicate that the HS pauses the cell cycle at two key points during the cell cycle in barley, first before DNA replication and second before mitosis. Such a double block also explains why there were no larger changes in the G1 and G2 nuclei proportion during the HS treatment. These findings are in agreement with the observations in tobacco BY-2 cells, where HS arrested the cell cycle in G1/S or G2/M phase depending on the time of its application (Jang et al., 2005).

In conclusion, we established the protocols for live semi-automated monitoring of barley root growth under various temperature conditions. This methodology can be applied universally to other cereal species after training the model. Increasing temperature efficiently accelerated barley root growth until it reached the level of HS, which started between 33°C and 35°C. At this temperature, root cells preserve a reduced ability to enter and finish mitotic division, though their shape is significantly affected. The usage of dedicated barley FMLs and cytology methods revealed that severe heat stress at 37°C arrested both mitotic division and cell cycle progression. Both processes restarted upon recovery at a lower temperature. A prominent phenotype was a synchronous entry into mitosis during the first post-stress cycle and absence of replicating cells during HS. This suggests that the replication and mitosis, two key events of the somatic cell cycle, are sensitive to HS, and their checkpoints might be critical for controlling the responses of barley roots to HS.

## Data Availability

The original contributions presented in the study are included in the article/**Supplementary Material**. Further inquiries can be directed to the corresponding author.
