# Supplementary material for: Heat stress suppresses DNA replication and mitosis in barley root apical meristems
Source: Front Plant Sci. 2025 Nov 27;16:1679234. doi: 10.3389/fpls.2025.1679234 (PMC12695828; doi:10.3389/fpls.2025.1679234)
Supplement: Supplementary file 1 [file DataSheet1.pdf]

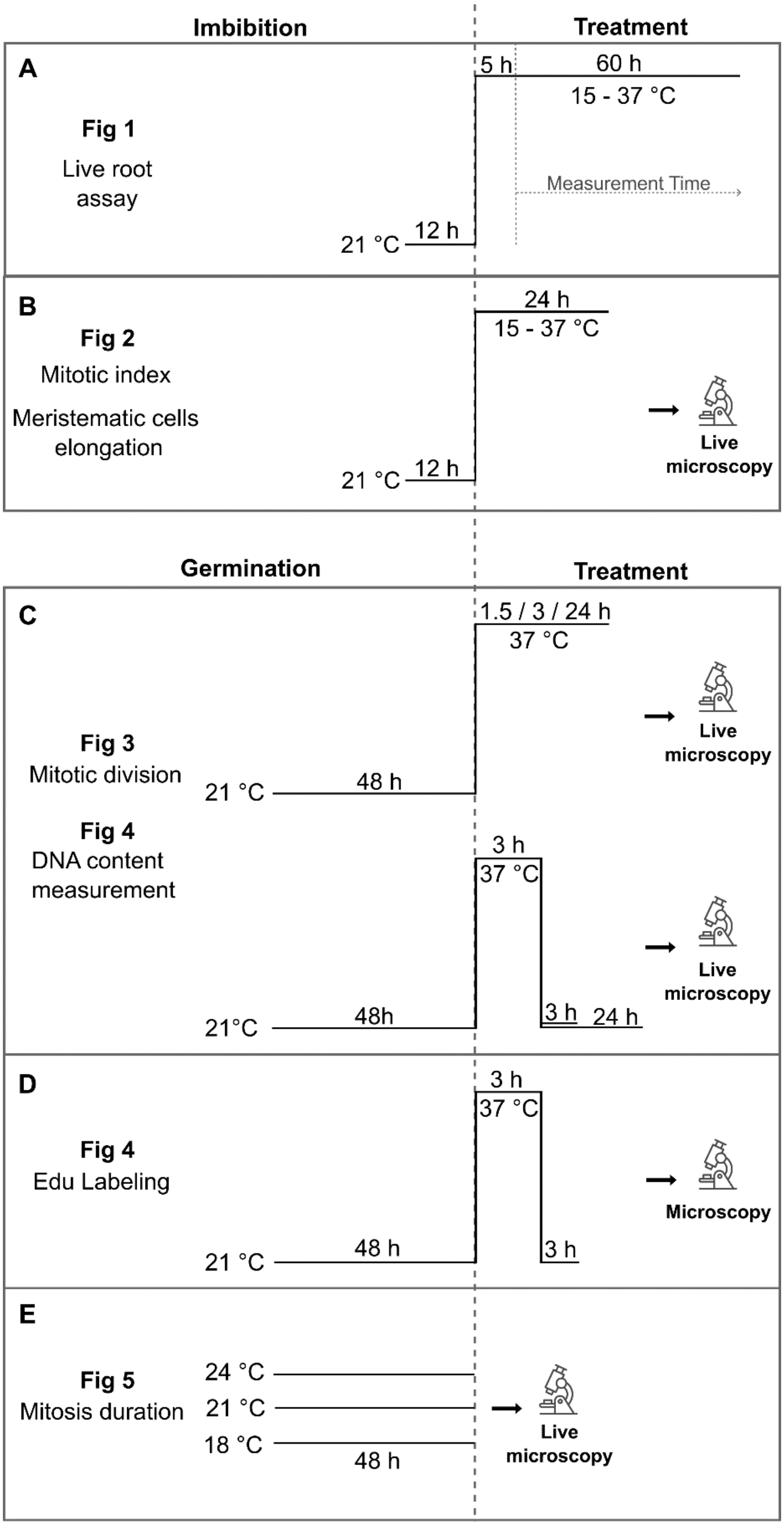

Supplementary Figure 1. Plant growth and treatment regimes used within the manuscript. A-E) An overview of growth conditions and treatment regimes used for the sample preparations in the individual experiments.

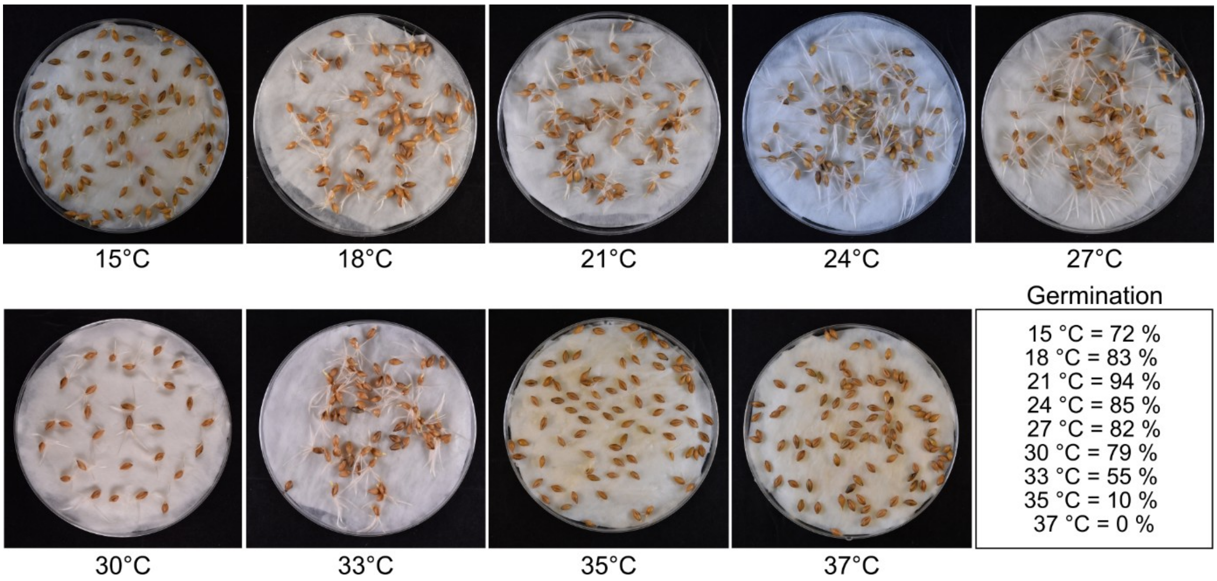

Supplementary Figure 2. Differences in barley seed germination under different treatment conditions. Representative pictures of Petri dishes with soaked, cold stratified (48 h, 4 °C) barley seeds after 2 days of germination. Germination index (GI) was calculated for 80 seeds per plate.

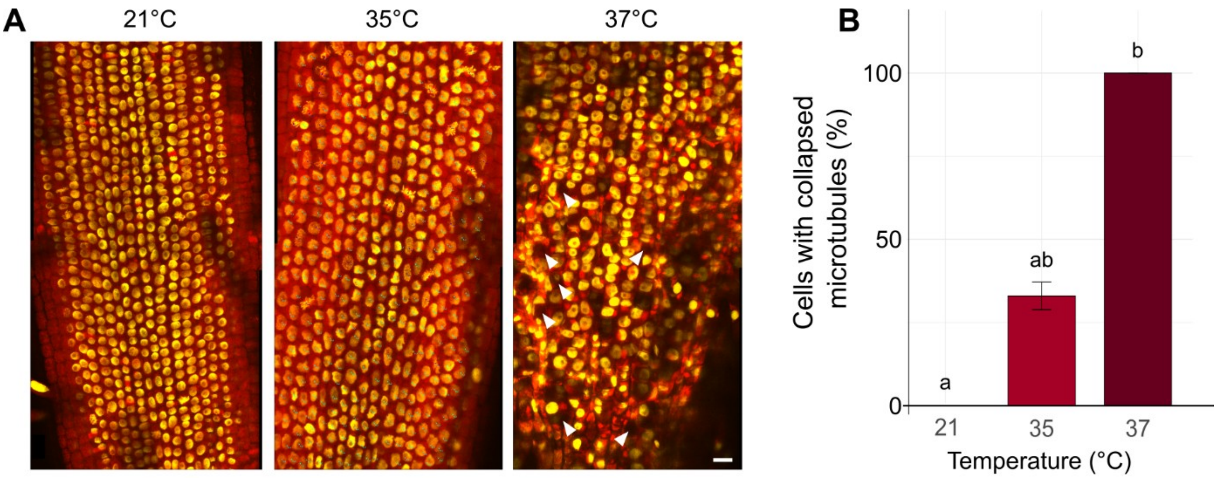

Supplementary Figure 3. Heat stress changes root anatomy and leads to the collapse of microtubule network. A) Representative photos of control (21 °C) and 24 h heat-stressed (35 °C and 37 °C) roots showing altered anatomy of cell files, cells, nuclei and collapsed microtubule network as indicated by EYFP-H2B (yellow) and mCherry-TUA3 (red). Positions with missing (or dead) cells after 37 °C treatment are visible as root parts without EYFP-H2B signals (white arrows). Scale bar = 20 μm. B) Percentage of cells with collapsed microtubular network. Error bars represent the standard error of the means among three individual plants and at least 100 cells per temperature. Statistical analysis was done using the Kruskal-Wallis statistical test and Dunn's post-hoc test; $P \leq 0.05$.
